# Finding Protein-Coding Genes through Human Polymorphisms

**DOI:** 10.1371/journal.pone.0054210

**Published:** 2013-01-22

**Authors:** Edward Wijaya, Martin C. Frith, Paul Horton, Kiyoshi Asai

**Affiliations:** 1 Graduate School of Frontier Sciences, University of Tokyo, Kashiwa, Japan; 2 Computational Biology Research Center, National Institute of Advanced Industrial Science and Technology (AIST), Tokyo, Japan; Inserm U869, France

## Abstract

Human gene catalogs are fundamental to the study of human biology and medicine. But they are all based on open reading frames (ORFs) in a reference genome sequence (with allowance for introns). Individual genomes, however, are polymorphic: their sequences are not identical. There has been much research on how polymorphism affects previously-identified genes, but no research has been done on how it affects gene identification itself. We computationally predict protein-coding genes in a straightforward manner, by finding long ORFs in mRNA sequences aligned to the reference genome. We systematically test the effect of known polymorphisms with this procedure. Polymorphisms can not only disrupt ORFs, they can also create long ORFs that do not exist in the reference sequence. We found 5,737 putative protein-coding genes that do not exist in the reference, whose protein-coding status is supported by homology to known proteins. On average 10% of these genes are located in the genomic regions devoid of annotated genes in 12 other catalogs. Our statistical analysis showed that these ORFs are unlikely to occur by chance.

## Introduction

Compilation of an accurate catalog of protein-coding genes encoded in human genomes is a critical step to fully understand the functional elements in human genomes. Many annotations of protein-coding genes have been published [1] and a plethora of gene finding software has been introduced [2]. Nevertheless, the task has remained as a great challenge [3]. As stated by Brent [4] the difficulty lies in the limitations of sequencing protocols, ways to combine the predicted genes and finally the limitations of human curators.

In response to these findings we also believe that there is an increasing amount of genomic evidence that may affect protein-coding gene detection, which has not been taken into account. Human polymorphism is one example of such evidence [5]. It has been suggested that such polymorphism affects protein-coding genes, and that they are responsible for various human diseases [6]–[8].

Several studies have tried to account for damaging polymorphism in known protein-coding genes. Such polymorphism could affect the amino acid sequence, alter protein function and contribute to disease. For example Ng and Henikoff [8] discovered that two known genes were mistakenly classified as pseudogenes because of mutations. Others analyzed predicted protein-coding genes and found known SNPs and insertions and deletions in them [9], [10]. However, to our knowledge no work has been done on the influence of polymorphism on the finding of novel genes.

**Figure 1:**
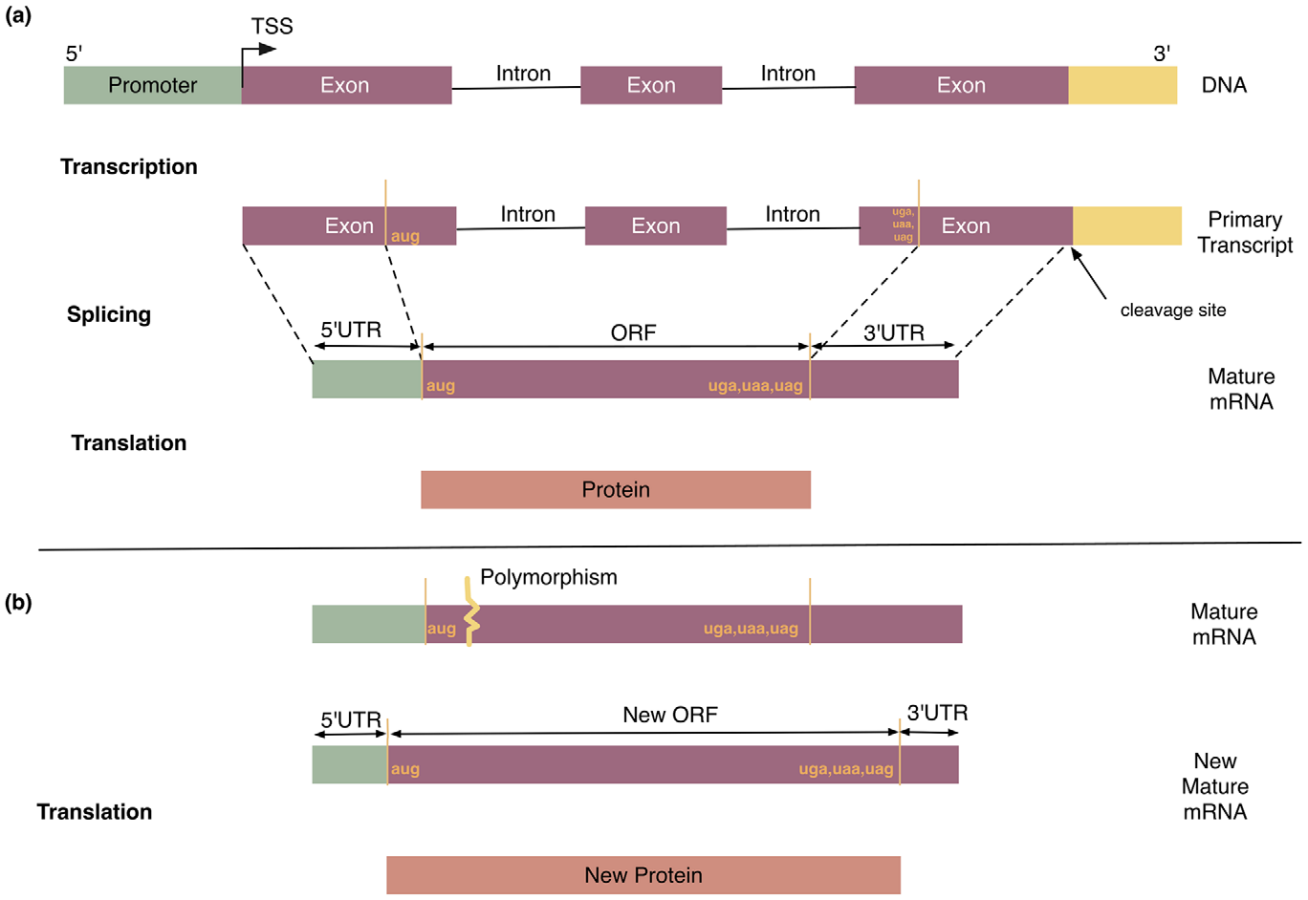
The standard model of genetic information transfer in molecular biology. Panel (a) shows the transfer begins with the DNA being transcribed into mRNA, and continues with protein being synthesized using information in mRNA as a template (translation). We investigate the effect of polymorphic modification of the mRNA. Panel (b) depicts how the new longer ORF was formed. The starting position of the new ORF in the mRNA is before that of original ORF. The new ORF may or may not overlap the original ORF, and if it does overlap, it is in different reading frame, so that the proteins are completely different.

In this article we provide a framework for finding new unannotated genes based on human polymorphism. We examine several types of polymorphism: SNPs, insertions, deletions, multiple nucleotide polymorphisms, and microsatellites. With this information we reconstruct the mRNAs and finally define the new open reading frames (ORFs). Figure 1a depicts the standard model for general transfers in biological sequence information. Figure 1b further illustrates the effect of polymorphism on the mature mRNA and its effect in defining the new ORF.

**Figure 2:**
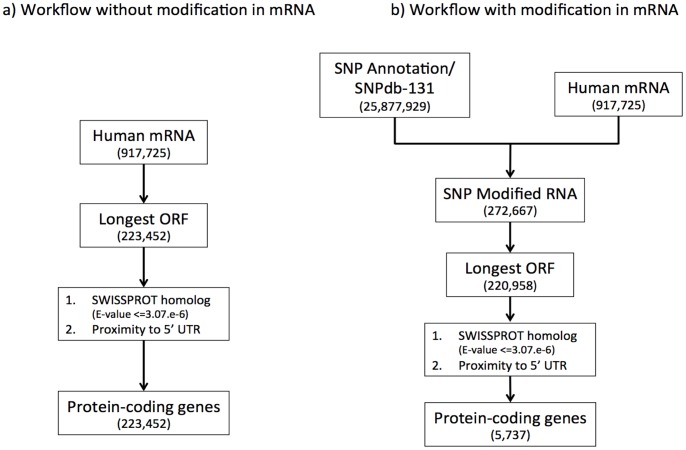
Workflows for finding protein-coding genes. Panel (a) describes the workflow of gene-finding without applying human polymorphism and (b) with human polymorphism. The values inside the brackets refer to the number mRNAs, ORFs and genes respectively. The final number of genes in workflow (b) refers to the genes where the ORFs change after modification, but in workflow (a) such change does not apply. For the second workflow (b) two main sources of data are used: human mRNA sequences and polymorphism data (dbSNP 131). Based on the polymorphism information we redefine the mRNA sequences. Out of the modified mRNA sequences we derived the longest ORFs. These ORFs are further refined by filtering them based on significant homology to Swiss-Prot and proximity to 5′ UTR. Finally we construct the genes from the refined ORFs.

Demonstrating definitively that a transcript encodes a protein is a difficult, time-consuming and expensive task. One approach is to synthesize the protein artificially, raise an antibody against it and use the antibody to test whether it is expressed in vivo. Even this does not discriminate functional proteins from translation noise caused by stochastic nonfunctional translation by ribosomes [11], [12]. On the other hand, proving that a transcript does not encode protein is an impossible task because the protein might only be expressed in very rare circumstances [13]. Thus we apply several bioinformatics criteria to improve the reliability of our findings: the ORF length, absence of repeats, E-value of homology to Swiss-Prot and shortness of 5′ untranslated region (UTR). Figure 2 illustrates two pipelines in parallel, those with and without polymorphism.

We identified 5,737 putative protein-coding genes that result from mRNA modified by human polymorphisms and have significant homology to known proteins. On average 10% of these genes are located in genomic regions unannotated by 12 other gene catalogs. A genomic coordinate list of these protein-coding genes is available as Table S1.

Among our other findings, we also discover that some of these novel genes are orthologous to genomic regions of other species where genes are annotated. Furthermore, we exhibit three examples of longest ORFs found after modification of the mRNA. They show significant homology to Swiss-Prot proteins and were not predicted as coding regions by any other gene finders. We cannot rule out that these mRNAs are fragments of longer mRNAs (i.e. artifacts), however the presence of supporting ESTs suggests that they are not.

## Results

We will first highlight the results by three examples. The genomic positions of the mRNA in these examples are provided in Table 1. There we also indicate the genomic positions of the longest ORF, the polymorphism that causes the modification, the length of 5′ UTR after modification, the E-value of homology to known proteins and the sequence orientation.

**Table 1:** Genomic positions of mRNA for the examples shown in Figure 3, 4, 5.

	AK124706	AK127273	AY129028
Chr. name	chrUn_gl000222	chr7	chr14
Strand	−	+	-
mRNA pos. in chr.	25,008–28,821	128,295,697–128,299,178	93,403,259–93,406,150
dbSNP ref.	rs66651466	rs71162510	rs8011546
ORF pos. after modification	28,249–28,585	128,296,043–128,296,637	93,404,946–93,405,303
ORF pos. before modification	27,864–28,166	128,298,398–128,298,944	93,404,502–93,404,811
5′ UTR	236	347	847
E-value of new ORF alignment to Swiss-Prot	3.21E-10	4.74E-50	1.66E-08

**Figure 3:**
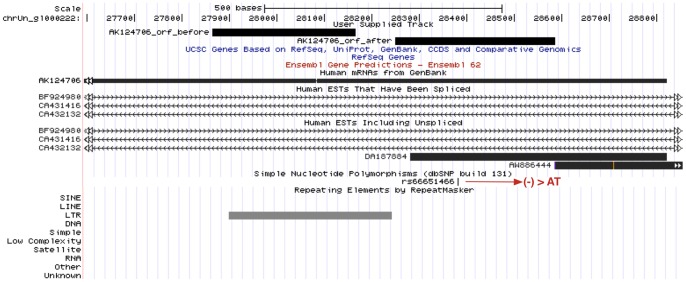
Modification by polymorphism of mRNA AK124706 and its ORFs. In the reference genome the modification is caused by an insertion (rs66651466) with ‘AT’ as the allele. The initial longest ORF before modification has length 302 bp. The new longest ORF has length 336 bp, and it aligns to Swiss-Prot Integrin beta-5 protein (Acc:P18084). Annotation of start/stop codon in the translation process and alleles that cause the change can be found in Figure 2.

Figure 3 shows a modification by polymorphism of mRNA AK124706. The new ORF is located upstream of the initial ORF. A repeating element overlaps the initial ORF, but none is found for the new ORF. There is also supporting evidence for transcription with the presence of EST (DA187884) at the 5′ end of the mRNA. It is aligned to Swiss-Prot *Integrin beta-5* protein (Acc: P18084) [14].

**Figure 4:**
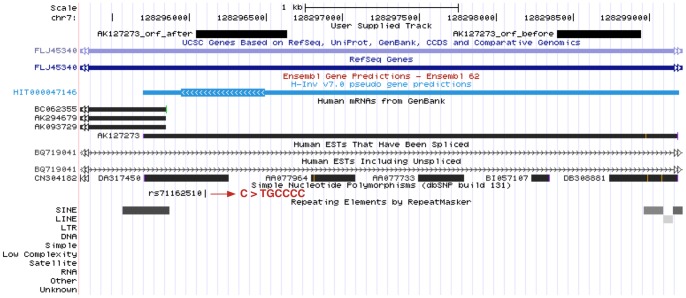
Modification by polymorphism of mRNA AK127273 and its ORFs. The initial longest ORF before modification has length 546 bp. The longest ORF after modification has length 594 bp. The polymorphism responsible for the modification is an in-del (rs71162510) which replaces the reference genome allele ‘C’ with ‘TGCCCC’.

Figure 4 shows modification of mRNA AK127273. The mRNA and ORFs are located on the forward strand of the chromosome. We note that the predicted longest ORF after modification does overlap a pseudogene (HIT00004716) predicted by H-Inv, however this pseudogene is on the reverse strand of the chromosome. The new ORF is 5′ of the initial ORF. We verified that an EST (DA317450) is present at the 5′ end of the mRNA. None of the existing gene finders predict a coding region in the same location as our new ORF, which aligns to Swiss-Prot protein *capicua homolog* (Acc: Q96RK0) [15].

**Figure 5:**
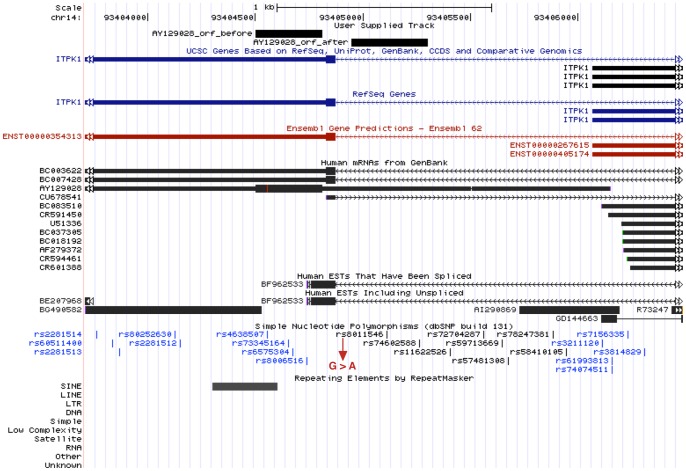
Modification by polymorphism of mRNA AY129028 and its ORFs. The initial longest ORF before modification has length 309 bp, and after has length 357 bp. The polymorphism that effects the modification is a SNP (rs8011546) which replaces the reference allele ‘G’ with ‘A’.

Figure 5 shows modification of mRNA AY129028. Although there is an existing coding region annotation in the mRNA, this just overlaps the initial ORF. The mRNA and ORFs are located on the reverse strand of the chromosome. Moreover, the initial ORF is located 3′ of the new ORF, and it overlaps a repeat element. This suggests that the ribosome would have to scan over the new ORF without translating it in order to reach the known ORF. Even though AY129028 looks like a fragment of pre-spliced mRNA, we found two ESTs (A1290869 and GD144663) at its 5′ end, evidence for a real transcript starting here. It appears in this figure that GD144663 starts at chr14:93,406,483–93,406,489 instead of chr14:93,406,109–93,406,489, however we believe the latter is correct. The alignment in the former position lacks a standard splice acceptor sequence, and alignment in the latter position would have 5 matches out of 7 (see Figure S1). The new ORF also aligns to Swiss-Prot protein FLJ4630 (Acc: Q6ZRH3) [14].

### Population Differences in ORF changes

Human populations from different parts of the world are surprisingly similar genetically. When averaged over all genes, the majority of genetic variation present in the human species can be found in any human ethnic group [16]. However the small level of differentiation among these groups is large enough for geneticists to make estimates on human diseases related to their ethnicity [17]. Results from large scale undertakings such as 1000 Genome [5] or HapMap Projects [18] for identifying human polymorphisms from diverse populations enable us to examine its significance in great detail.

We investigate the percentage of ORF-changing alleles for eleven different populations. These populations include: European-derived individuals from Utah pedigrees (CEU), Han Chinese in Beijing (CHB), Japanese in Tokyo (JPT) and Yoruba in Ibadan Nigeria (YRI). In addition to that we also included the expanded populations: African Ancestry in SouthWestern United States (ASW), Chinese Ancestry in Metropolitan Denver, CO, US (CHD), Gujarati Indians in Houston, TX (GIH), Luhya in Webuye, Kenya (LWK), Mexican Ancestry in Los Angeles, CA, US (MEX), Masai in Kinyawa, Kenya (MKK) and Toscani in Italia (TSI).

**Figure 6:**
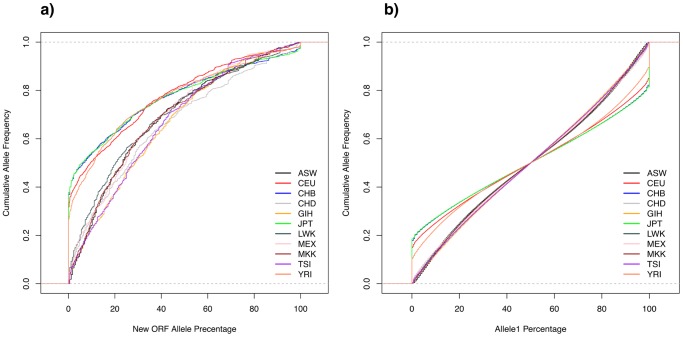
Cumulative allele frequency from 11 populations. In panel (a) we plot the allele percentage of new ORFs and (b) allele percentage of all HapMap data in UCSC Genome Browser. The percentage (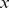-axis) in panel (b) is based on Allele1, chosen arbitrarily.

The normalized cumulative frequency of allele percentage can be seen in Figure 6a. We observed that there are slight differences in the cumulative frequency on seven populations: ASW, CHD, GIH, LWK, MEX, MKK, TSI. It is interesting to note that some populations are closely grouped together, e.g. ASW-MKK and CHB-JPT.

Four of the populations (CEU, CHB, JPT, and YRI) stand out in Figure 6a. In these populations around 40% of the new ORFs are extremely rare. In order to understand this, we plot the frequency of all alleles in each population (Figure 6b) directly from the UCSC HapMap data, regardless whether they cause new ORFs or not. The same four populations also stand out as having rare alleles. The reason presumably is an artifact of how the data was collected. These four populations belong to the earlier phase of HapMap project than the other seven populations [19].

For all the ORFs that underwent change, we discover that the majority of them (54%) appear in all 11 populations and around 23% in 4 populations (CEU, CHB, JPT and YRI). In Supplementary Material S1 Section 2 we listed the detailed breakdown of the occurrences of ORFs in every population and additionally in Table S2 we have included lists of ORFs and their occurrence in every population.

### Genes and mRNA Counts Affected By Polymorphism

In this work, an ORF is said to be new if it shares no same-frame codons with the initial ORF before modification in mRNA. This implies that these proteins could be completely distinct from one another. In Table 2 we list the numbers of human ORFs and genes affected by polymorphism.

**Table 2:** ORFs and genes that changed after modification.

	ORFs	Genes
Unchanged	202,232	26,491
Changed	18,726	5,737

An ORF is said to be new or undergo changes if it shares no same-frame codons with the initial ORF before modification in the mRNA. The genes are constructed by merging the ORFs (from different mRNAs) that overlap in the same strand of a chromosome.

We also looked at the types of polymorphism that cause ORF modification. The table in Supplementary Material S1 Section 3 indicates five types of polymorphisms: deletion (DLT), insertion (INS), insertion/deletion (IND) microsatellite (MIC), multiple nucleotide polymorphism (MNP), and single nucleotide polymorphism (SNP). We found that the primary source of modification that causes ORF change is SNP (79%) followed by insertion (3%), deletion (6%), insertion/deletion (1%), MNP (0.01%) and microsatellite (0.01%).This is consistent with the fact that SNP is the most common in the dbSNP database.

Among all the ORFs and genes, there are 8% a of them are affected by polymorphism. In order to verify whether the new ORF could be significantly affected by other polymorphisms, we perform a further experiment. From the new mRNA after initial modification we re-apply polymorphisms onto it (2nd modification). Although the second modification does change the counts of the disrupted ORFs, the effect is small (Supplementary Material S1 Section 5). One possible future direction following this result is to investigate the cancelling effect of the above second modification.

### Frequency of Random ORFs

We performed a control procedure where the chromosomal positions of polymorphism are randomized. The mRNAs are then modified with these false polymorphisms. Then we applied the same software and parameterization as described in the Method section for determining the longest ORF. Table 3 shows that the number of changes in ORF and gene after modification by random polymorphism is smaller than those caused by real polymorphism. In Supplementary Material S1 Section 1, we detail further the differences of these ORF predictions with respect to their overlap with known genes of other species.

**Table 3:** Effect of randomization in ORFs and genes prediction.

Polymorphisms type	ORFs	Genes
Real	18,726	5,737
Random	3,004	1,330

Similar to the previous table, the figures refer to the number of ORFs that changed after modification with real and random polymorphisms. These figures are reported after validating the ORFs through Swiss-Prot homology.

### Comparison With Other Gene Catalogs

In general gene prediction approaches can be classified into two categories: sequence similarity based searches and structure or signal based searches. The similarity search approach is based on finding similarity of ESTs (expressed sequence tags) and proteins to the input genome. On the other hand signal based searches rely on information such as motifs, splice sites, start and stop codons to identify the genes. Our method belong to both categories, where we treat the polymorphisms as signal and validation through Swiss-Prot proteins as similarity based approach. We examine the discrepancy of the genes found by our methods with the existing gene finders.

There are fourteen gene finders used by the UCSC Genome Browser to annotate the human genome. They are: Acembly [20], CCDS [21], Ensembl [22], GeneID [23], Genscan [24], KnownGene [25], H-InvDB [26], NSCAN [27], RefGene [28], SGP [29], Vega [30] and Xenoref [28]. We compile the number of our predicted genes that were not found and found by these gene finders in Table 4. The majority of our genes do have overlap, i.e. partially share genomic location, with those genes from other finders. Our finding should complement the results from existing gene finders. On average 10% of our novel coding genes have zero overlap with any given one of these gene finders. It was with H-InvDB genes where we found the greatest number of gene overlaps, around 5% of our genes have no overlap with it.

**Table 4:** Number of new genes after modification by polymorphism with and without overlap with each of 12 other gene sets.

Gene Finder	No Overlap	With Overlap
acembly	365	5,372
ccdsGene	713	5,024
ensGene	473	5,264
geneid	541	5,196
genscan	449	5,288
hinv70Coding	284	5,453
knownGene	452	5,285
nscanGene	521	5,216
refGene	521	5,216
sgpGene	498	5,239
vegaGene	1,762	3,975
xenoRefGene	622	5,115

### Gene Comparison With Other Species

Recently there is a growing interest in the origin of human protein coding genes [31]. It was suggested that novel genes regularly appear from messenger RNAs of ancestral genes, and such novel genes significantly affect the evolution of cellular, physiological, morphological, behavioral and reproductive phenotypic traits [32]–[34].

We compared our novel protein coding human genes subject to polymorphism with their counterpart genes in 25 other genomes. For these genomes we find their genes based on the annotation given by Ensembl gene prediction software [22]. Table 5 shows the number of new protein coding genes discovered by our method that overlap and do not overlap with genes in each of the species. Surprisingly, we discovered that there are some overlaps between predicted genes and the known genes of these species. Moreover, we can distinguish between mammals and non-mammals from the table. The mammals have more overlaps (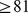) than non-mammals.

**Table 5:** Number of new genes after human polymorphism modification that overlap with other species' genes and not found by Ensembl gene finder (ensGene).

	With Overlap	No Overlap
chicken (galGal3)	65	408
medaka (oryLat2)	68	405
zebrafish (danRer7)	71	402
zebrafinch (taeGut1)	72	401
panda (ailMel1)	76	397
tetraodon (tetNig2)	77	396
frog (xenTro2)	78	395
lizard (anoCar1)	78	395
fugu (fr2)	80	393
stickleback (gasAcu1)	81	392
cat (felCat3)	81	392
pig (susScr2)	83	390
orangutan (ponAbe2)	86	387
chimpanzee (panTro2)	87	386
marmoset (calJac3)	89	384
rhesus (rheMac2)	89	384
rabbit (oryCun2)	90	383
guinea pig (cavPor3)	92	381
horse (equCab2)	93	380
elephant (loxAfr3)	94	379
mouse (mm9)	94	379
cow (bosTau4)	96	377
rat (rn4)	97	376
dog (canFam2)	101	372
opossum (monDom5)	104	369

### Gene Ontology

Discovery of new genes will be useful if we can elucidate their roles in various biological domains. Gene ontology (GO) provides such a framework. We use Uniprot to estimate gene ontologies of our newly defined ORFs based on their homologs in Swiss-Prot, because Uniprot provides high-quality gene ontology annotation for proteins from multiple species [35].

We tabulate three categories of gene product (molecular function, cellular function and biological process) where the ORFs are over-represented (Table 6, 7, 8). The ontologies are assigned using the web based application GOrilla [36].

**Table 6:** Molecular function gene ontology of new ORFs after modification.

Description	P-Value	FDR q-value	Enrichment (N,B,n,b)
ATP binding	1.49E-33	5.71E-30	1.46 (16846,1442,5262,659)
adenyl ribonucleotide binding	5.77E-33	1.11E-29	1.45 (16846,1466,5262,666)
binding	9.74E-33	1.25E-29	1.09 (16846,11419,5262,3897)
adenyl nucleotide binding	2.80E-32	2.69E-29	1.45 (16846,1475,5262,667)
catalytic activity	1.12E-27	8.63E-25	1.19 (16846,5141,5262,1909)
protein binding	1.40E-25	8.98E-23	1.14 (16846,7073,5262,2519)
purine ribonucleoside triphosphate binding	1.90E-25	1.04E-22	1.35 (16846,1776,5262,751)
purine ribonucleotide binding	4.93E-25	2.36E-22	1.35 (16846,1806,5262,760)
ribonucleotide binding	4.93E-25	2.10E-22	1.35 (16846,1806,5262,760)
purine nucleotide binding	1.67E-24	6.40E-22	1.34 (16846,1818,5262,762)
nucleotide binding	5.94E-21	2.07E-18	1.27 (16846,2318,5262,921)
nucleoside phosphate binding	6.92E-21	2.21E-18	1.27 (16846,2319,5262,921)
small molecule binding	8.24E-21	2.43E-18	1.26 (16846,2485,5262,978)
ion binding	1.70E-19	4.65E-17	1.19 (16846,3833,5262,1426)
cation binding	2.72E-19	6.95E-17	1.19 (16846,3825,5262,1422)
metal ion binding	4.41E-19	1.06E-16	1.19 (16846,3754,5262,1397)
kinase activity	4.11E-18	9.27E-16	1.47 (16846,756,5262,347)
phosphotransferase activity, alcohol group as acceptor	1.79E-17	3.82E-15	1.48 (16846,704,5262,325)
protein kinase activity	7.02E-17	1.42E-14	1.51 (16846,592,5262,280)
transferase act. transferring phosphorus containing grp.	7.95E-17	1.52E-14	1.42 (16846,875,5262,387)

Ranked top 20 terms according to the P-value of overrepresentation against the background set. Last column with ‘Enrichment (N, B, n, b)’ is defined as follows: 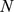 is the total number of genes, 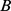 is the total number of genes associated with the corresponding GO term (description), 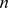 is the number of genes in the target set, 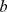 is the number of genes in the intersection. Enrichment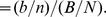 Note that the total number of target genes (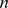) in the last column could be less or equal to the number of input genes. This is because GOrilla normalised the input gene names with its gene database.

**Table 7:** Biological process gene ontology of new ORFs after modification.

Description	P-Value	FDR q-value	Enrichment (N,B,n,b)
macromolecule modification	1.23E-13	1.51E-10	1.23 (16846,1977,5262,762)
phosphorylation	5.86E-13	6.48E-10	1.43 (16846,628,5262,280)
Phosphate containing compound metabolic process	9.70E-13	9.75E-10	1.37 (16846,798,5262,342)
phosphorus metabolic process	9.70E-13	8.94E-10	1.37 (16846,798,5262,342)
protein phosphorylation	1.14E-12	9.73E-10	1.44 (16846,575,5262,259)
cellular protein modification process	3.48E-12	2.75E-09	1.23 (16846,1884,5262,721)
protein modification process	3.48E-12	2.57E-09	1.23 (16846,1884,5262,721)
regulation of biological process	1.63E-11	1.12E-08	1.08 (16846,7733,5262,2615)
protein metabolic process	6.10E-11	3.97E-08	1.16 (16846,2921,5262,1061)
regulation of biological quality	3.45E-10	2.12E-07	1.19 (16846,2036,5262,759)
transmem.eceptor protein tyrosine kinase sig. pathway	4.16E-10	2.42E-07	1.42 (16846,489,5262,217)
response to stimulus	6.81E-10	3.77E-07	1.10 (16846,5534,5262,1901)
regulation of cellular process	8.46E-10	4.46E-07	1.08 (16846,7330,5262,2470)
enzyme linked receptor protein signaling pathway	2.26E-09	1.14E-06	1.34 (16846,658,5262,276)
cellular protein metabolic process	3.41E-09	1.64E-06	1.17 (16846,2348,5262,856)
cellular response to stimulus	4.72E-09	2.18E-06	1.12 (16846,4170,5262,1453)
organelle organization	5.27E-09	2.33E-06	1.20 (16846,1654,5262,621)
regulation of response to stimulus	6.76E-09	2.87E-06	1.17 (16846,2110,5262,774)
cellular component organization	7.02E-09	2.88E-06	1.14 (16846,3139,5262,1115)
Peptidyl tyrosine phosphorylation	8.21E-09	3.24E-06	2.17 (16846,59,5262,40)

Ranked top 20 terms according to the P-value of overrepresentation against the background set.

**Table 8:** Cellular component gene ontology of new ORFs after modification.

Description	P-Value	FDR q-value	Enrichment (N,B,n,b)
cell part	2.57E-32	3.30E-29	1.08 (16846,12147,5262,4108)
intracellular part	6.23E-28	4.00E-25	1.08 (16846,11593,5262,3922)
organelle	2.22E-18	9.52E-16	1.11 (16846,7819,5262,2703)
intracellular organelle	3.61E-18	1.16E-15	1.11 (16846,7802,5262,2696)
cytoplasmic part	2.28E-17	5.85E-15	1.12 (16846,6470,5262,2268)
cytosol	1.01E-16	2.16E-14	1.24 (16846,2261,5262,878)
cytoplasm	1.03E-14	1.88E-12	1.16 (16846,3850,5262,1398)
Membrane bounded organelle	3.36E-14	5.39E-12	1.10 (16846,6897,5262,2377)
intracellular membrane bounded organelle	3.74E-14	5.33E-12	1.10 (16846,6892,5262,2375)
organelle part	8.10E-10	1.04E-07	1.09 (16846,6066,5262,2070)
intracellular organelle part	9.22E-10	1.08E-07	1.09 (16846,5980,5262,2042)
nucleus	1.36E-09	1.46E-07	1.11 (16846,4598,5262,1597)
non-membrane-bounded organelle	1.55E-09	1.53E-07	1.19 (16846,1920,5262,715)
Intracellular non-membrane bounded organelle	1.55E-09	1.42E-07	1.19 (16846,1920,5262,715)
nuclear part	6.90E-08	5.91E-06	1.15 (16846,2412,5262,866)
cytoplasmic vesicle part	7.30E-07	5.86E-05	1.37 (16846,390,5262,167)
cell junction	1.60E-06	1.21E-04	1.26 (16846,694,5262,274)
Golgi apparatus	5.61E-06	4.00E-04	1.26 (16846,629,5262,248)
cell projection	8.08E-06	5.46E-04	1.22 (16846,819,5262,313)
nucleoplasm	1.10E-05	7.05E-04	1.21 (16846,912,5262,344)

Ranked top 20 terms according to the P-value of overrepresentation against the background set.

In the initial experiment we used a target set of gene names from Uniprot that have homology to our predicted ORFs, and a background set of gene names from Uniprot that have homology with longest ORFs before modification by polymorphism. In this experiment we wish to show what is overrepresented in the new ORFs with respect to old ORFs. We found that in the molecular function category, *ATP binding is the most overrepresented term with significant P-value (1.49E-33)*. For the biological process category we found that *macromolecule modification* is the most overrepresented term with significant P-value (1.23E-13). Finally for the cellular component category we found terms with significant P-value (2.57E-32) related to *cell part*.

We also performed another experiment where we apply gene names from Uniprot that have homology to old ORF and our newly predicted ORFs as target sets separately. All human proteins from Uniprot are used as background set. With this experiment we want to show if the polymorphism does change the GO assignment, and whether we can find more significant terms compared to the old ORFs. The results (Supplementary Material S1 Section 6) showed that polymorphism does indeed change the GO assignment. Moreover, the P-values of GO terms for ORFs after modification are more significant than those before modification.

## Conclusion

In this work we describe our findings on the importance of human polymorphism in determining protein-coding genes. Here we indicate that the human population harbors a number of genes that have been missed by previous analyses focusing on one reference genome alone. In our approach we employ strict bioinformatics criteria: ORF length, absence of repeats, E-value of ORF homology to Swiss-Prot, and length of 5′ UTR to verify our findings. We demonstrate that from the new protein-coding genes there were some not found by other gene-finder algorithms, but located in genomic regions where genes are annotated in other species.

The UCSC Genome Browser also continuously updates their data especially with regards to newer human genome assemblies, introduction of new genomes, new gene predictors and annotation of polymorphism. Along with this information, in the short-term when more complete SNP catalogs, frequency data and more distant species will be made available; we hope to explore further the impact of human variations in gene finding. It would be useful for example, to probe for the differences between newly predicted genes and known annotated genes by looking at their sequence similarity in addition to their genomic regions.

And eventually there will be data that include haplotype combinations, more RNAs as well as full-length transcripts. To adapt to these future changes, we believe it would be helpful to make available a gene database based on our approach. A dynamic browsing function for the location of ORFs before and after modification would intuitively convey our results.

## Materials and Methods

### Source of sequence and polymorphism data

For consistency, all our information described below is obtained from a single resource: the UCSC Genome Browser. First of all, the human mature mRNA sequences from GenBank [37] were used as the source for deriving ORFs. The sequences were obtained from http://hgdownload.cse.ucsc.edu/goldenPath/hg19/bigZips/mrna.fa.gz. In total there are 917,725 mRNA sequences. To construct the genes we utilize the annotation of mRNA location in the human genome. It can be downloaded from http://hgdownload.cse.ucsc.edu/goldenPath/hg19/database/all_mrna.txt.gz.

We applied human polymorphism from the dbSNP build131 database [38] for reconstructing the mRNAs. In total we considered 25,877,929 variations. The annotation of these variants on the human genome (hg19) was obtained from http://hgdownload.cse.ucsc.edu/goldenPath/hg19/database/snp131.txt.gz. The variants consist of: SNP - single nucleotide polymorphism, MNP- multiple nucleotides polymorphism, microsatellite - variation in forms of short tandem repeats, insertion - the polymorphism as insertions relative to the reference assembly, deletion - the polymorphism as deletion relative to the reference assembly and in-del - insertion/deletion. In-del is a special class defined by dbSNP. We compare the length of the reference allele to the length(s) of observed alleles; if the reference allele is shorter than all other observed alleles, then ‘in-del’ will be considered as ‘insertion’. Likewise, if the reference allele is longer than all other observed alleles, it will be considered as ‘deletion’. The table in Supplementary Material S1 Section 4 shows the overall frequencies of the above mentioned polymorphisms.

### Defining mRNAs and ORFs from polymorphism

The reconstruction of mRNA is done by mutating, inserting or deleting the nucleotides in the mRNA according to the variant alleles in dbSNP, based on the positions of the variants and mRNA in the human genome (hg19). Lacking information on haplotypes (i.e. combinations of polymorphisms), we applied each polymorphism on its own. Thus, it is possible that some haplotypes that we considered do not exist in any individual. For each newly reconstructed mRNA we obtained the longest ORF using getorf from EMBOSS [39], with options -min 300 -norev -find 1. We used 300 bp nucleotide as minimum length for ORFs prediction as suggested by many studies that this is an appropriate threshold [40], [41]. Our approach is also based on the standard view of mRNA that it only encodes one distinct protein.

The sequence of triplet codons in ORFs beginning with ATG and ending with a stop codon represents the protein. Here we only look at ORFs with 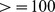 codons, because three out of sixty-four codons encode stops and ORFs greater than 100 codons are unlikely to appear by chance in non-coding sequences of average composition [13]. We define genes by merging the ORFs from different mRNAs that overlap in the same strand of a chromosome. Figure 2 exhibits the procedure for identifying novel coding genes through polymorphism. Notice that in our procedure, there is no re-alignment of the modified mRNA to the human genome involved. For the purpose of determining the genomic loci of exons of new mRNAs, we incorporate the mRNA annotation provided by the UCSC Genome Browser. In this annotation the coordinates of the mRNAs in the genome are specified. Such information enables us to convert the exon locations in new mRNAs into exon locations in the genome.

We also find supporting evidence to show that our newly predicted ORFs are translated into proteins. As a first criterion, we selected ORFs that aligned to the manually curated Swiss-Prot protein database with alignment score 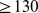. This scoring threshold corresponds to significant homology (E-value 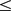
3.07e-6). At the end of this step we obtained 5,737 protein-coding genes. The majority of the ORFs have low E-values (0) as can be seen from their cumulative distribution in Supplementary Material S1 Section 4. This procedure will allow us to reduce erroneous translation of frameshifted or non-coding nucleotide sequences. The alignment was done using LAST [42]. It contains two sub-programs: lastdb and lastal. We indexed the protein database with lastdb with parameter -p to indicate that the sequences are amino acids and -c to soft-mask lower case letters. The actual alignment was done using lastal with parameter -e 130 which sets the minimum score for gapped alignments to 130. The E-value was computed using the lastex program that comes in the package. Both the translated ORFs and Swiss-Prot proteins were repeat masked using TANTAN [43] before performing the alignment.

As a final criterion we select ORFs based on proximity to the mRNA 5′ end. It has been suggested that the ribosome scanning mechanism favors short 5′ UTRs, whereas long 5′ UTRs are likely to contain encumbrances, e.g. upstream start codons or secondary structure elements [44], [45].

### Allele Frequency

The percentage of alleles is obtained from the count of individuals who are homozygous and heterozygous for the observed alleles. This information is available from the UCSC Genome Browser HapMap annotation: http://hgdownload.cse.ucsc.edu/goldenPath/hg19/database/hapmapSnps.txt.gz. Following their nomenclature, the zygote counts are defined as follows: 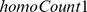 and 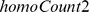 are counts of individuals who are homozygous for the first allele and second allele respectively; *heteroCount* is the count of individuals who are 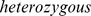.

Subsequently we define counts of first of and second alleles (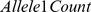 and 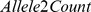):
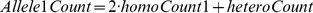


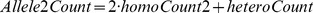



Finally the allele percentage can be obtained as:
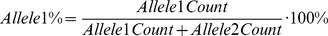



These percentages are then computed with respect to our predicted ORFs, by looking at the correspondence of modifying variants in our method and the HapMap annotation above.

### Comparison with Other Gene Catalogs

There are three sources of information involved in comparing our result to other gene catalogs: 1) list of the new ORFs with their positions in mRNA, 2) alignment of human mRNA with the human genome, and 3) fourteen gene annotations of the human genome. The primary information we employed from these annotations are the predicted starting and ending positions of the exons. We obtained the last two sources from the UCSC Genome Browser.

The key idea in these steps is to compare the overlaps with respect to the genomic location of our predicted ORFs and those predicted by other finders. Initially, we located the positions of the new ORFs in the human genome. We can do that by transferring the positions of new ORFs in mRNA with respect to alignment of human mRNA to the human genome. Since the annotations of all other catalogs already provide the genomic location of the exons, we can easily find their overlap or non-overlap with the modified new ORFs.

### Gene Comparison with Other Species

For this task we use the following data sources: 1) list of new ORFs obtained after modification and their corresponding position in the mRNA, 2) alignment of human mRNA with the human genome, 3) gene prediction for human genome (hg19) and all other 25 species provided by Ensembl software [22], 4) human genome alignment with other species. We obtained data sources 2 to 4 from the UCSC genome browser.

Similarly to the previous section, we defined overlap based on the genomic location of new ORFs and gene location of other species aligned to human genome. Hence, the first step we did was to find the positions of the new ORFs in the human genome. This can be done by transferring the positions of data source (1) using data source (2). The next crucial step is to find the location of exons of all other species predicted by Ensembl that align to the human genome. The key data source for this step is (3) and (4). Finally, the overlap and non-overlap of new ORFs in human and all other species can be determined.

### Gene Ontology

In finding over-represented gene ontology terms resulting from the newly predicted genes after polymorphism, we applied GOrilla (http://cbl-gorilla.cs.technion.ac.il/), a web-based application. We used *two unranked* lists of genes as the option. We performed two types of experiments for the analysis:

1. For the first experiment, the target list contains 5,630 gene names from Uniprot that have homology to our 18,726 predicted ORFs. The background set consists of 46,961 gene names from Uniprot that have homology with ORFs before modification by polymorphism.
2. In the second experiment, we use all 71,124 human gene names from Uniprot as background set. Then *separately* we use 5,630 gene names from ORFs after and 46,961 gene names from ORFs before modification by polymorphisms as target set.

In both of the above experiments when an ORF has multiple hits to the Uniprot gene names, we only took gene name with highest E-value.

GOrilla uses the minimum hypergeometric score (mHG) statistic for computing the P-value. In total there are 11,109 GO terms used in our computation. To correct the P-value for multiple testing, we further compute the FDR q-value [46]. It is computed as:
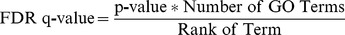



## Supporting Information

Figure S1 GD144643 alignment to hg19.
(PDF)

Figure S2 Effect of polymorphism in the translation of the 3 examples (AK124706, AK127273 and AY129028).
(PDF)

Table S1 List of new genes after modification of mRNA by polymporhisms.
(BED)

Table S2 List of new ORFs categorized for 11 populations.
(TXT)

Supporting Information S1 Gene Ontology data used for experiment as described in the method section.
(ZIP)

Supplementary Material S1 Alignment of EST GD144663 and chr14.
(PDF)
