# Supplementary material for: Finding Protein-Coding Genes through Human Polymorphisms
Source: PLoS One. 2013 Jan 22;8(1):e54210. doi: 10.1371/journal.pone.0054210 (PMC3551959; doi:10.1371/journal.pone.0054210)
Supplement: Figure S1 — GD144643 alignment to hg19. (PDF) [file pone.0054210.s001.pdf]

Alignment of GD144663 and chr14:93406109-93406489

Matching bases in cDNA and genomic sequences are colored blue and capitalized. Light blue bases mark the boundaries of gaps in either sequence (often splice sites).

cDNA GD144663

```
CATGGAGCTC AAGTTTGTTA TGTCATACTC CCAGTCCTTA AAAGACGTTT  50
AATAAAACAG CTTTACCTTT TAAAAATAAA A
```

Genomic chr14 (reverse strand):

```
ttgaaaaagt gaaaaaggct tccgggctgt cctctgccca gtgagatgga  93406540
ggacgctaga gaaagtgctg agtgtcccga gagaggcccc cgagccagtg  93406490
CATGGAGgtc cttcggcctg gctcagctgg gctgcaggat gcccactttg  93406440
aggagggagg cacagggctt gggcgagggg cagaggccat cagaactgcc  93406390
cggctttttt ggaaactgag gacccaacaa ctaaccacgt ttacacgact  93406340
tgagttttga accccgatta atgtctgtac gtcacctttc ctagttctga  93406290
ccctgagccc tggggaacag gaaagcgtgg ctggcctctt gcactgcttt  93406240
gtctccaaaa taaactactg aaatcaaacc gcatttcaca cgtgtttaca  93406190
catgttgCTC AAGTTTGTTA TGTCATACTC CCAGTCCTTA AAAGACGTTT  93406140
AATAAAACAG CTTTACCTTT TAAAAATAAA Atttcaatcc cagtctgcct  93406090
caggtacggg tgggggtgtg cagcgtgact cctggctgtg ttcctgtacc  93406040
gtttgtggcc tccggggtct tgcgcgtcca g
```

Side by Side Alignment

```
00000001 catggag 00000007
<<<<<<<< ||||||| <<<<<<<<
93406489 catggag 93406483


00000008 ctcaagtttgttatgtcatactcccagtccttaaaagacgtttaataaaa 00000057
<<<<<<<< |||||||||||||||||||||||||||||||||||||||||||||||||| <<<<<<<<
93406182 ctcaagtttgttatgtcatactcccagtccttaaaagacgtttaataaaa 93406133

00000058 cagctttaccttttaaaaataaaa 00000081
<<<<<<<< |||||||||||||||||||||||| <<<<<<<<
93406132 cagctttaccttttaaaaataaaa 93406109
```

Source: UCSC Genome Browser
