# Supplementary material for: Finding Protein-Coding Genes through Human Polymorphisms
Source: PLoS One. 2013 Jan 22;8(1):e54210. doi: 10.1371/journal.pone.0054210 (PMC3551959; doi:10.1371/journal.pone.0054210)
Supplement: Figure S2 — Effect of polymorphism in the translation of the 3 examples (AK124706, AK127273 and AY129028). (PDF) [file pone.0054210.s002.pdf]

EFFECT OF POLYMORPHISM IN TRANSLATION OF EXAMPLE ORFS FROM THE mRNAS

Start and stop codon are indicated in red font.

After AK124706

```
                                         (-)>at rs66651466
        201 cactctctctcatctgaaatatttccattttccatcatgtttccatcagcctgcactcctctcacttcccgttatcttgttcgcttccacactaatatct 300

F1        3 H  S  L  S  S  E  I  F  P  F  S  I  M  F  P  S  A  C  T  P  L  T  S  R  Y  L  V  R  F  H  T  N  I  S 36

        301 ctaaacaggtaactgtgttcctcaaggacttaaaagcacttaacaacagcttaaaacttaggttctcactctttagcaaggacttgaagacttttaatga 400

F1       37  K  Q  V  T  V  F  L  K  D  L  K  A  L  N  N  S  L  K  L  R  F  S  L  F  S  K  D  L  K  T  F  N  D  69

        401 ttcagctcctgttcacaaatctaaccagactttttcccacatcaaccctgaacagaagctaagttccactcatatacaaaatctttcaattttcctgaat 500

F1       70  S  A  P  V  H  K  S  N  Q  T  F  S  H  I  N  P  E  Q  K  L  S  S  T  H  I  Q  N  L  S  I  F  L  N   102

        501 aaaaaagattatgtagccatcacccataatctgactccagaactctgtttctgcaccttcctgtcaaactcataataagtctccatattagtctattctc 600

F1      103 K  K  D  Y  V  A  I  T  H  N  L  T  P  E  L  C  F  C  T  F  L  S  N  S  *  *  V  S  I  L  V  Y  S  H 8
```

Before AK124706

```
        601 gctgctaatatggacatgcccaaggctgggtaatttataaagaaaaataagtttagtttaatggactcacagttccacatggctggggagacctcacaat 700

F2        9 A  A  N  M  D  M  P  K  A  G  *  F  I  K  K  N  K  F  S  L  M  D  S  Q  F  H  M  A  G  E  T  S  Q  S 23

        701 catggcagaaggcaaagatcaggtcttacacggcagcagacgagagagcatgtgtaggggaacttccctccatacaaccatcagatctcatgagacttat 800

F2       24  W  Q  K  A  K  I  R  S  Y  T  A  A  D  E  R  A  C  V  G  E  L  P  S  I  Q  P  S  D  L  M  R  L  I  56

        801 tcactatcataagaacaacacagtcctcatgattcaattacctcccccaggttcctcccacaatatgtgggaattatgggaacaacaattcgagatttg 900

F2       57  H  Y  H  K  N  N  T  V  L  M  I  Q  L  P  P  P  R  F  L  P  Q  Y  V  G  I  M  G  T  T  I  R  D  L   89

        901 ggtgaggacacagccaaaccatatcagtctcataataaggaccaaaggccaagccttccctaagtcgtccttaagagactcatgcagatttcataatttg 1000

F2       90 G  E  D  T  A  K  P  Y  Q  S  H  N  K  D  Q  R  P  S  L  P  *  V  V  L  K  R  L  M  Q  I  S  *  F  A 2
```

After AK127273

```
                                                          c>tgcccc rs71162510
     301 gggggctgaggaggtgtgagcccctgccaggaacccctgcccagaccatgcccctggcccacaggcccctgatgtctgcgtccagcgaggcctccagtg 400
F1     2 G  G  *  G  G  V  S  P  C  Q  E  P  P  A  Q  T  M  P  L  A  H  R  P  L  M  S  A  S  S  E  A  S  S  G 31
     201 atctcaaaaaaaaaaaaaaacaaaaacaaaaacttgaggcctggcctcatgctccccttccatccccacttccatgggtccaagctgccttggctgagga 300
F1    41  S  Q  K  K  K  K  T  K  T  K  T  *  G  L  A  S  C  S  P  S  I  P  T  S  M  G  P  S  C  L  G  *  G   1
     301 gggggctgaggaggtgtgagcccctgccaggaacccctgcccagaccatgtacttggcccacaggcccctgatgtctgcgtccagcgaggcctccagtg 400
F1     2 G  G  *  G  G  V  S  P  C  Q  E  P  P  A  Q  T  M  Y  L  A  H  R  P  L  M  S  A  S  S  E  A  S  S  G 31
     401 gcgtcagcaTGCCCCtttgtgtggaggaacgtggaaccttgctctgtggctgtgttctcctggtactctgtccccttcctgacccctccctgcagctatg 500
F1    32  V  S  M  P  L  C  V  E  E  R  G  T  L  L  C  G  C  V  L  L  V  L  C  P  L  P  D  P  S  L  Q  L  C  64
     501 tgaggtccagcaacctgccagtcactcagtggcctccaaccagagcaaagaacctgccaagtcagcagctgttgctcatgagtgtccaccaggtgggaca 600
F1    65  E  V  Q  Q  P  A  S  H  S  V  A  S  N  Q  S  K  E  P  A  K  S  A  A  V  A  H  E  C  P  P  G  G  T   97
     601 gggagtgctgaccctgggtggcccccctggagccacctgccctgaaagcccagggcccgcaaccccacacactttgggggtggtggaacctggtaaaagct 700
F1    98 G  S  A  D  P  G  W  P  P  G  A  T  C  P  E  S  P  G  P  A  T  P  H  T  L  G  V  V  E  P  G  K  S  S 131
     701 cacctcccaccatggaggaggagccctgggcccctcaggggagtccctgctggacagtgagacagagaatgaccatgatgatgctttcctctccatcatg 800
F1   132  P  P  T  M  E  E  E  P  W  A  P  Q  G  S  P  C  W  T  V  R  Q  R  M  T  M  M  M  L  S  S  P  S  C  164
     801 tctcctgacacccagttgcctctaccacccagatgatgtcaggcccagtccctcagtgccctgcgcaaggaacaggactcatcttctgagaaggatggat 900
F1   165  L  L  T  P  S  C  L  Y  H  P  D  D  V  R  P  S  P  S  V  P  C  A  R  N  R  T  H  L  L  R  R  M  D   197
     901 gcagccccaacaaatgggacaaggaccacatccggtggcccatgagtggcggtcatgatcttcagcaagcggcaccaggccctggcagggcgcaccaggg 1000
F1   198 A  A  P  T  N  G  T  R  T  T  S  G  G  P  *  V  A  V  M  I  F  S  K  R  H  Q  A  L  A  G  R  T  R  V 19
```

Before AK127273

```
      2701 gacatgatggagaggaaagcatcatcatggtcattctctgtctcactgtccagcagggactcccctgaggggcccagggctcctcctccatggtgggagg 2800
F2      13 D  M  M  E  R  K  A  S  S  W  S  F  S  V  S  L  S  S  R  D  S  P  E  G  P  R  A  P  P  P  W  W  E  V 46
      2801 tgagcttttaccaggttccaccacccccaaagtgtgtggggttgcgggccctgggctttcagggcaggtggctccaggggccacccagggtcagcactc 2900
F2      47   S  F  Y  Q  V  P  P  P  P  K  C  V  G  L  R  A  L  G  F  Q  G  R  W  L  Q  G  A  T  Q  G  Q  H  S  79
      2901 cctgtcccacctggtggacactcatgagcaacagctgctgacttggcaggttctttgctctggttggaggccactgagtgactggcaggttgctggacct 3000
F2      80  L  S  H  L  V  D  T  H  E  Q  Q  L  L  T  W  Q  V  L  C  S  G  W  R  P  L  S  D  W  Q  V  A  G  P   112
      3001 cacatagctgcagggaggggtcaggaaggggacagagtaccaggagaacacagccacagagcaaggttccacgttcctccacacaaacatgctgacgcca 3100
F2     113 H  I  A  A  G  R  G  Q  E  G  D  R  V  P  G  E  H  S  H  R  A  R  F  H  V  P  P  H  K  H  A  D  A  T 146
      3101 ctggaggcctcgctggacgcagacatcaggggcctgtgggccaagtacatggtctgggcaggggggttcctggcaggggctcacacctcctcagccccctc 3200
F2     147   G  G  L  A  G  R  R  H  Q  G  P  V  G  Q  V  H  G  L  G  R  G  F  L  A  G  A  H  T  S  S  A  P  S  179
      3201 ctcagccaaggcagcttggacccatggaagtggggatggaaggggagcatgaggccaggcctcaagtttttgtttttgtttttttttttttttgagatgc 3300
F2     180  S  A  K  A  A  W  T  H  G  S  G  D  G  R  G  A  *  G  Q  A  S  S  F  C  F  C  F  F  F  F  L  R  C   16
```

After AY129028

g>a rs8011546

801 ctcgcctgtatcacctgagtcaggcttatgcctgggcggggcagatgactgtggcatcggggtgtggccaggctggcctgaggcagcagaggctgggcct 900

F2 25 R L Y H L S Q A Y A W A G Q **M** T V A S G C G Q A G L R Q Q R L G L 57

901 ttgtcaaggacagctgtacttctctgcctcccggtcaccaggctggaggggtcgtgctcctttagggcagccagcacccagctcaaggtctctgaatcca 1000

F2 58 C Q G Q L Y F S A S R S P G W R G R A P L G Q P A P S S R S L N P 90

1001 atggcccatgagccacagctcgggaaggagaaggagctgccggggatcccaccgggtgaatgttcagagtttgtttgtttgtttgtttgtttttgagga 1100

F2 91 M A H E P Q L G K E K E L P G I P P G E C S E F V C L F V C F L R S 124

1101 gtcttgctctgttgccaggctggagtgcagtggtgcgatcttggctcactgcaacctacgcctcccgagttcaagcaattctcctgcctcagcctcccaa 1200

F2 125 L A L L P G W S A V V R S W L T A T Y A S R V Q A I L L P Q P P K 157

1201 gtagctgggactacaggcgcccaccaccatgtccagctaattttgtatttttagtagagatggggtttcaccatgttggccaggatggtgtcgatctct 1300

F2 1 * L G L Q A P T T M S S * F L Y F * * R W G F T M L A R M V S I S 14

Before AY129028

1301 aaggctggaagaccgactgaagtaggaagctgtcacatgtttgagccaactgagcaaagcagctgttgcctgccagacagccaccccaagaccaagggag 1400

F3 1 K A G R P T E V G S C H **M** F E P T E Q S S C C L P D S H P K T K G A 34

1401 ccagcctgtcgtttgtccttctagcctggaagaccagttcttggctcttgggcactcacattggccactggccgcaactgctgaatggcttcactaatgt 1500

F3 35 S L S F V L L A W K T S S W L L G T H I G H W P Q L L N G F T N V 67

1501 ccctggggtctgtatctgccctctcttctattccctagaaactctggccgggcatggcggctcacgcctgtcatcccagcacttagggaggccgaggcag 1600

F3 68 P G V C I C P L F Y S L E T L A G H G G S R L S S Q H L G R P R Q 100

1601 gtggatcacaaggtcaagagatcgacaccatcctggccaacatggtgaaaccccatctctactaaaaatacaaaaattagctggacatggtggtgggcgc 1700

F3 101 V D H K V K R S T P S W P T W * N P I S T K N T K I S W T W W W A P 18

ALIGNMENT OF EXAMPLE ORFS TO SWISSPROT

AFTER MODIFICATION

```
E-value=3.21e-10
s sp|P18084|ITB5_HUMAN          156 50 + 79   NIRSLGTKLAEEMRKLTSNFRLGFGSFVDKDISPFSYTAPRYQTNPCIGY
AK124706_after                   20  50 + 112  NISKQVTVFLKDLKALNNSLKLRFSLF-SKDLKTFNDSAPVHKSNPTFSH

E-value= 4.74e-50
s sp|Q96RK0|CIC_HUMAN           0 138 + 1608 MYSAHRPLMPASSAASRGLGMFVWTNVEprsvavfpwhslvpflapsqpdpsvqpseaqqpashpvasnqs
s AK127273_after                0 133 + 198  MPLAHRPLMSASSEASSGVSMPLC--VEERGTLLCGCVLLV--LCP-LPDPSLQLCEVQQPASHSVASNQS

                                             kepaesaavaherppggtgsadperppgatcpespgpgpPHPLGVVESGKgppptteeeasgppGEP
                                             KEPAKSAAVAHECPPGGTGSADPGWPPGATCPESPGPATPHTLGVVEPGKSSPPTMEEEPWAPQGSP
E-value= 1.66e-08
s sp|Q6ZRH3|YA014_HUMAN        124 29 + 153 PGWSAVARSRLTATSASRVEAILLPQPPE
s AY129028_after               90 29  + 119 PGWSAVVRSWLTATYASRVQAILLPQPPK
```

BEFORE MODIFICATION

```
E-value=47.6
s sp|Q6NH32|Y1313_CORDI     190 26 + 299 RDTDLLIDVRFLPNPFWVPELRPFRG
s AK056566_before           40 26 + 127  RDEWLYANLRVPSLPFWVPETDPRKG

E-value= 2.12e-06
s sp|Q9H8N2|CA136_HUMAN 12 31 + 187 HSGRQEGACAGELPFIKPSDLMRHIHYHENS
s AK124706_before       22 31 + 100 YTAADERACVGELPSIQPSDLMRLIHYHKNN

E-value= 5.13e-131
s sp|Q6ZSN7|YG040_HUMAN 0 182 + 182 MMERKASSWSFSVSLSSRDSPEGPRAPppwwevsfyqvppppKCVGLRALGFQGRWLQGATQGQHSLSHLVDTHEQQLLT
s AK127273_before       0 182 + 182 MMERKASSWSFSVSLSSRDSPEGPRAPPPWWEVSFYQVPPPPKCVGLRALGFQGRWLQGATQGQHSLSHLVDTHEQQLLT

                                   WQVLCSGWRPLSDWQVAGPHIAAGRGQEGDRVPGEHSHRARFHVPPHKHADATGGLAGRRHQGPVGQVHGLGRGFLAGAH
                                   WQVLCSGWRPLSDWQVAGPHIAAGRGQEGDRVPGEHSHRARFHVPPHKHADATGGLAGRRHQGPVGQVHGLGRGFLAGAH

                                   TSSAPSSAKAAWTHGSGDGRGA
                                   TSSAPSSAKAAWTHGSGDGRGA
```

```
E-value=27.9
s sp|Q95PZ0|CSN6_CAEEL 321 14 + 426 RFKSQHLGRPQQAD
s AY129028_before       76 14 + 103 RLSSQHLGRPRQVD
```
