# Supplementary material for: Finding Protein-Coding Genes through Human Polymorphisms
Source: PLoS One. 2013 Jan 22;8(1):e54210. doi: 10.1371/journal.pone.0054210 (PMC3551959; doi:10.1371/journal.pone.0054210)
Supplement: Supplementary Material S1 — Alignment of EST GD144663 and chr14. (PDF) [file pone.0054210.s006.pdf]

Supplementary Material

1. Simulation based on randomized variations.

We randomized the chromosomal positions of all the polymorphism annotations. Then we re-ran our procedure (with and without Swiss-Prot) using these randomized variants to obtain new ORFs. An ORF is said to be new if it shares no same-frame codons with the 'old ORF'.

Below is the summary:

Number of changes in ORFs after modification with real polymorphisms

	>=1 same-frame codons	No same-frame codons
Before Swiss-Prot	64,213	22,203
After Swiss-Prot	58,357	18,726

Number of changes in ORFs after modification with random polymorphisms

	>=1 same-frame codons	No same-frame codons
Before Swiss-Prot	15,612	5,930
After Swiss-Prot	9,390	3,004

Note that the number of ORF changes that share no same-frame codons with old ORFs are smaller than those that shares with >=1 same-frame codons. Also, when we consider random polymorphism, the number of changes after Swiss-Prot is smaller than real polymorphism (3,004/5,930 versus 18,726/22,203). The latter indicates that most of the real SNPs coincide more with start/stop codon than random SNPs.

We also looked at how many of these random novel ORFs overlap with other species. The table below exhibits the comparison. Table below showed that the number of overlaps from the ORFs generated by random SNP is fewer than non-random.

	Overlap Non Random	Overlap Random
chicken (galGal3)	65	10
medaka (oryLat2)	68	10
zebrafish (danRer7)	71	9
zebrafinch (taeGut1)	72	8
panda (ailMel1)	76	10

tetraodon (tetNig2)	77	10
frog (xenTro2)	78	9
lizard (anoCar1)	78	9
fugu (fr2)	80	10
stickleback (gasAcu1)	81	10
cat (felCat3)	81	10
pig (susScr2)	83	10
orangutan (ponAbe2)	86	10
chimpanzee (panTro2)	87	9
marmoset (calJac3)	89	10
rhesus (rheMac2)	89	9
rabbit (oryCun2)	90	11
guinea pig (cavPor3)	92	11
horse (equCab2)	93	11
elephant (loxAfr3)	94	11
mouse (mm9)	94	11
cow (bosTau4)	96	11
rat (rn4)	97	11
dog (canFam2)	101	11
opossum (monDom5)	104	11

2. The occurrence of new ORFs in 11 populations.

Table below shows the number of occurrences (frequency) of ORFs that appear in exactly 1 to 11 populations. For example there are 2,912 ORFs that appear in exactly 4 populations. It shows that majority (54%) of these ORFs do appear in all the 11 populations and around 23% appear in 4 populations (CEU, CHB, JPT and YRI).

Number of Populations	Frequency of New ORFs	Percentage from total
1	96	0.80%
2	84	0.70%
3	214	1.79%
4	2,812	23.47%
5	292	2.44%
6	216	1.80%
7	316	2.64%
8	326	2.72%
9	346	2.89%
10	801	6.69%
11	6,479	54.07%
Total	11,962	100%

We compute the frequency of ORFs that are affected by polymorphism by populations. Below is the table.

Population	New ORFS
ASW	8,236
CEU	11,620
CHB	11,594
CHD	7,409
GIH	7,707
JPT	11,574
LWK	8,288
MEX	7,968
MKK	8,146
TSI	7,907
YRI	11,674

It must be noted that not all the polymorphism data in dbSNP131 are population specific. Out of 25,877,929 polymorphism data in only 4,164,537 are population specific.

3. Number of gene change by type of polymorphism.

The polymorphisms included are deletion (DLT), indel (IND), insertion (INS), microsatellite (MIC), multiple nucleotide polymorphism (MNP) and single nucleotide polymorphism (SNP). The last row shows the initial overall frequency of the polymorphism in the annotation file (snp131.txt).

	DLT	IND	INS	MIC	MNP	SNP
(a) Changed	1,186	225	2,427	2	2	14,848
(b) Changed Normalized (a/c)	6.81E-04	3.55E-04	9.07E-04	4.39E-04	2.57E-06	7.10E-04
(c) Overall	1,740,295	634,302	2,674,748	4,552	777,234	20,746,798

4. Cumulative count of the E-value from the alignment of new ORFs to Swiss-Prot protein

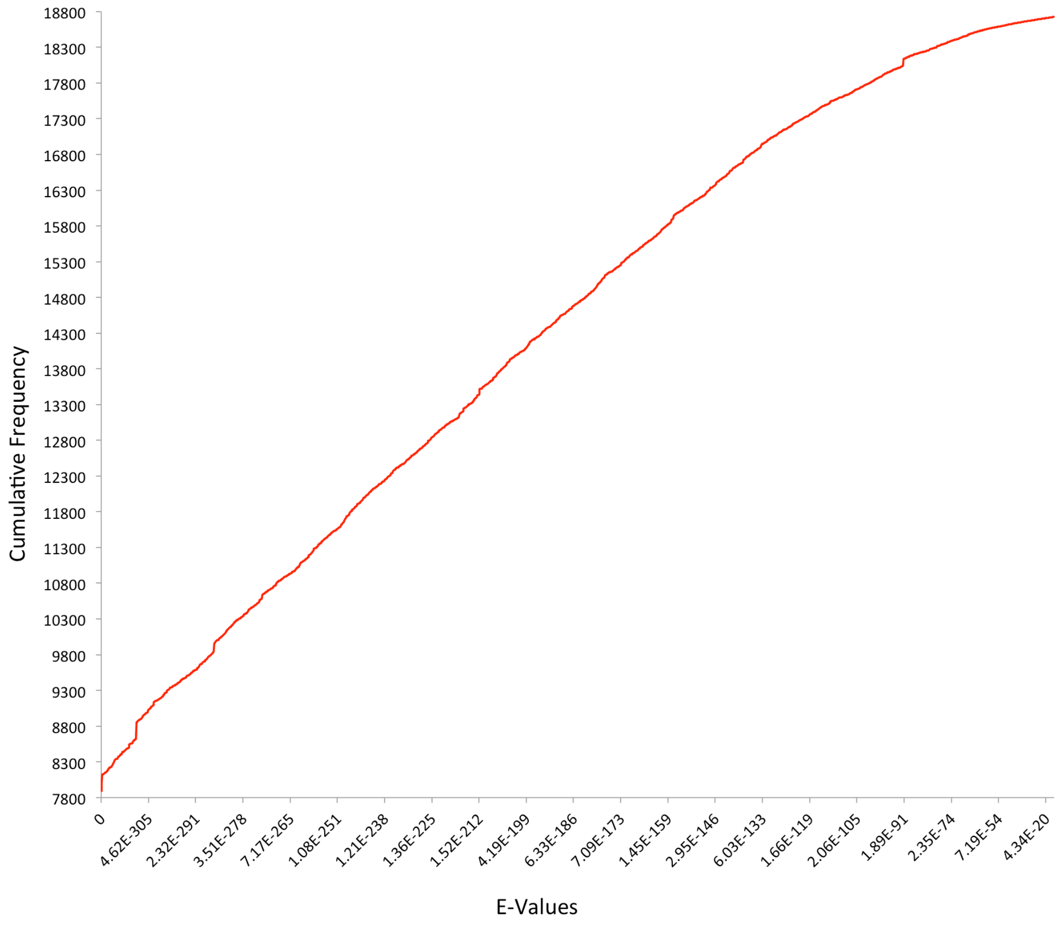

5. Effect of second SNP remodification of new mRNA.

In order to verify whether the new ORF could be significantly affected by other polymorphisms, we perform a further experiment. From the new mRNA after initial modification we reapply polymorphisms onto it (2nd modification). The table below shows the number of ORFs derived from the initial mRNA versus ORFs derived from second modification.

	mRNA (1st modification)	Newer mRNA (2nd modification)
Unchanged	202,232	202,172
Changed	18,726	18,786

Although the second modification does change the counts of the disrupted ORFs, the difference is not significant.

6. Comparison of GO terms of ORFs before and after modification.

In this experiment the genes from the human proteome in Uniprot are used as the background set. Secondly we apply new ORFs and old ORFs as target set separately. The results showed that polymorphism does indeed change the GO assignment.

We find it is surprising that the GO terms of ORFs before modification are significant. Presumably, this is because the gene names are filtered through Swiss-prot, which contains primarily human curated genes. The P-value of GO terms for ORFs after modification are lower (more significant) than those before modification. Furthermore, the enrichment of the GO terms is higher for all for those after modification.

The total number of GO terms from all 3 categories is 11,109.

Cellular Component (Before Modification vs. All Genes)

Description	P-value	FDR q-value	Enrichment (N,B,n,b)
membrane-bounded organelle	5.64E-13	7.24E-10	1.01 (17184,6973,16844,6897)
intracellular membrane-bounded organelle	6.09E-13	3.91E-10	1.01 (17184,6968,16844,6892)
organelle	1.61E-12	6.89E-10	1.01 (17184,7913,16844,7819)
intracellular organelle	2.08E-12	6.68E-10	1.01 (17184,7896,16844,7802)
nucleus	2.84E-11	7.29E-09	1.01 (17184,4640,16844,4598)
plasma membrane	7.25E-10	1.55E-07	1.01 (17184,3269,16844,3244)
cytoplasm	7.67E-07	1.41E-04	1.01 (17184,3892,16844,3850)
membrane	2.60E-06	4.18E-04	1.01 (17184,5393,16844,5324)
intrinsic to plasma membrane	4.07E-06	5.82E-04	1.02 (17184,1062,16844,1058)
integral to plasma membrane	8.31E-06	1.07E-03	1.02 (17184,1021,16844,1017)
cytosol	1.56E-05	1.82E-03	1.01 (17184,2282,16844,2261)
nuclear part	2.82E-05	3.02E-03	1.01 (17184,2436,16844,2412)
plasma membrane part	2.17E-04	2.15E-02	1.01 (17184,1865,16844,1847)
Golgi apparatus	2.66E-04	2.44E-02	1.02 (17184,631,16844,629)
cell part	2.67E-04	2.29E-02	1.00 (17184,12362,16844,12147)

Cellular Component (After Modification vs. All Genes)

Description	P-value	FDR q-value	Enrichment (N,B,n,b)
cell part	1.11E-33	1.43E-30	1.09 (17184,12362,5262,4108)
intracellular part	5.77E-29	3.71E-26	1.09 (17184,11801,5262,3922)
organelle	9.28E-21	3.98E-18	1.12 (17184,7913,5262,2703)
intracellular organelle	1.60E-20	5.15E-18	1.12 (17184,7896,5262,2696)
cytoplasmic part	4.34E-18	1.11E-15	1.13 (17184,6578,5262,2268)
cytosol	5.23E-18	1.12E-15	1.26 (17184,2282,5262,878)
membrane-bounded organelle	2.53E-16	4.65E-14	1.11 (17184,6973,5262,2377)
intracellular membrane-bounded organelle	2.86E-16	4.60E-14	1.11 (17184,6968,5262,2375)

cytoplasm	4.02E-16	5.74E-14	1.17 (17184,3892,5262,1398)
nucleus	3.85E-11	4.95E-09	1.12 (17184,4640,5262,1597)
non-membrane-bounded organelle	6.21E-10	7.25E-08	1.20 (17184,1948,5262,715)
intracellular non-membrane-bounded organelle	6.21E-10	6.65E-08	1.20 (17184,1948,5262,715)
organelle part	2.00E-09	1.98E-07	1.09 (17184,6199,5262,2070)
intracellular organelle part	2.39E-09	2.19E-07	1.09 (17184,6112,5262,2042)
nuclear part	1.00E-08	8.60E-07	1.16 (17184,2436,5262,866)
cytoplasmic vesicle part	3.98E-07	3.20E-05	1.38 (17184,394,5262,167)
cell junction	5.26E-07	3.98E-05	1.28 (17184,699,5262,274)
Golgi apparatus	1.44E-06	1.02E-04	1.28 (17184,631,5262,248)
cell projection	2.62E-06	1.77E-04	1.24 (17184,825,5262,313)
nucleoplasm	5.47E-06	3.51E-04	1.22 (17184,923,5262,344)

Biological Process (Before Modification vs. All Genes)

Description	P-value	FDR q-value	Enrichment (N,B,n,b)
cellular process	2.58E-18	2.87E-14	1.01 (17184,11815,16844,11658)
regulation of cellular process	8.70E-18	4.83E-14	1.01 (17184,7402,16844,7330)
regulation of biological process	1.52E-16	5.61E-13	1.01 (17184,7815,16844,7733)
primary metabolic process	6.78E-16	1.88E-12	1.01 (17184,7213,16844,7140)
biological regulation	9.90E-16	2.20E-12	1.01 (17184,8262,16844,8170)
regulation of metabolic process	9.15E-13	1.69E-09	1.01 (17184,4595,16844,4557)
regulation of primary metabolic process	1.29E-12	2.05E-09	1.01 (17184,4093,16844,4062)
regulation of macromolecule metab. proc.	1.32E-12	1.83E-09	1.01 (17184,3950,16844,3921)
macromolecule metabolic process	6.77E-12	8.36E-09	1.01 (17184,5804,16844,5745)
regulation of nitrogen comp. metabolic prc.	8.07E-12	8.97E-09	1.01 (17184,3253,16844,3232)
biological_process	8.39E-12	8.48E-09	1.00 (17184,14122,16844,13894)
regulation of nucleobase-containing compound metabolic process	8.72E-12	8.07E-09	1.01 (17184,3172,16844,3152)
regulation of cellular metabolic process	9.96E-12	8.51E-09	1.01 (17184,4174,16844,4140)
regulation of gene expression	4.95E-11	3.93E-08	1.01 (17184,3144,16844,3123)
positive regulation of biological process	3.39E-10	2.51E-07	1.01 (17184,3318,16844,3293)
regulation of macromolecule biosynth. Prc.	4.47E-10	3.10E-07	1.01 (17184,3008,16844,2987)
regulation of cellular biosynthetic process	4.95E-10	3.23E-07	1.01 (17184,3149,16844,3126)
regulation of biosynthetic process	9.74E-10	6.01E-07	1.01 (17184,3178,16844,3154)
regulation of cellular macromolecule biosynthetic process	1.21E-09	7.07E-07	1.01 (17184,2945,16844,2924)
regulation of RNA metabolic process	1.32E-09	7.32E-07	1.01 (17184,2790,16844,2771)

Biological Process (After Modification vs. All Genes)

Description	P-value	FDR q-value	Enrichment (N,B,n,b)
biological_process	3.56E-25	3.96E-21	1.05 (17184,14122,5262,4558)
primary metabolic process	3.09E-22	1.72E-18	1.13 (17184,7213,5262,2497)
cellular process	3.68E-22	1.36E-18	1.07 (17184,11815,5262,3885)
metabolic process	2.74E-21	7.6E-18	1.12 (17184,7829,5262,2681)
macromolecule metabolic process	3.83E-18	8.5E-15	1.14 (17184,5804,5262,2025)

biological regulation	1.29E-17	2.38E-14	1.10 (17184,8262,5262,2786)
cellular metabolic process	1.75E-15	2.78E-12	1.11 (17184,7102,5262,2410)
cellular macromolecule metabol. proc.	2.22E-15	3.08E-12	1.14 (17184,5317,5262,1849)
macromolecule modification	1E-14	1.24E-11	1.25 (17184,1995,5262,762)
regulation of biological process	9.71E-14	1.08E-10	1.09 (17184,7815,5262,2615)
phosphorylation	1.77E-13	1.78E-10	1.44 (17184,634,5262,280)
phosphate-containing compound metabolic process	2.19E-13	2.03E-10	1.39 (17184,805,5262,342)
phosphorus metabolic process	2.19E-13	1.87E-10	1.39 (17184,805,5262,342)
protein phosphorylation	3.3E-13	2.62E-10	1.46 (17184,580,5262,259)
cellular protein modification process	3.43E-13	2.54E-10	1.24 (17184,1901,5262,721)
protein modification process	3.43E-13	2.38E-10	1.24 (17184,1901,5262,721)
regulation of cellular process	6.41E-12	4.19E-09	1.09 (17184,7402,5262,2470)
protein metabolic process	9.83E-12	6.07E-09	1.17 (17184,2958,5262,1061)
transmembrane receptor protein tyrosine kinase signaling pathway	9.11E-11	5.33E-08	1.44 (17184,491,5262,217)
response to stimulus	1.53E-10	8.47E-08	1.10 (17184,5621,5262,1901)

Molecular Function (Before Modification vs. All Genes)

Description	P-value	FDR q-value	Enrichment (N,B,n,b)
molecular_function	1.57E-47	6.03E-44	1.01 (17184,14657,16844,14478)
binding	1.96E-42	3.77E-39	1.01 (17184,11523,16844,11417)
small molecule binding	1.61E-19	2.06E-16	1.02 (17184,2488,16844,2485)
nucleoside phosphate binding	3.36E-19	3.23E-16	1.02 (17184,2321,16844,2319)
nucleotide binding	3.44E-19	2.64E-16	1.02 (17184,2320,16844,2318)
organic cyclic compound binding	4.85E-19	3.11E-16	1.01 (17184,5136,16844,5102)
heterocyclic compound binding	4.95E-19	2.72E-16	1.01 (17184,5135,16844,5101)
protein binding	5.60E-19	2.69E-16	1.01 (17184,7137,16844,7072)
purine nucleotide binding	8.49E-16	3.62E-13	1.02 (17184,1819,16844,1818)
purine ribonucleotide binding	1.10E-15	4.24E-13	1.02 (17184,1807,16844,1806)
ribonucleotide binding	1.10E-15	3.85E-13	1.02 (17184,1807,16844,1806)
purine ribonucleoside triphosphate binding	2.12E-15	6.77E-13	1.02 (17184,1777,16844,1776)
catalytic activity	1.15E-13	3.39E-11	1.01 (17184,5186,16844,5141)
metal ion binding	1.73E-13	4.76E-11	1.01 (17184,3779,16844,3754)
cation binding	1.79E-13	4.59E-11	1.01 (17184,3851,16844,3825)
ion binding	5.35E-13	1.28E-10	1.01 (17184,3860,16844,3833)
adenyl nucleotide binding	1.34E-12	3.03E-10	1.02 (17184,1476,16844,1475)
adenyl ribonucleotide binding	1.62E-12	3.46E-10	1.02 (17184,1467,16844,1466)
ATP binding	2.69E-12	5.44E-10	1.02 (17184,1443,16844,1442)
receptor activity	4.06E-11	7.81E-09	1.02 (17184,1447,16844,1445)

Molecular Function (After Modification vs. All Genes)

Description	P-value	FDR q-value	Enrichment (N,B,n,b)
biological_process	3.56E-25	3.96E-21	1.05 (17184,14122,5262,4558)
primary metabolic process	3.09E-22	1.72E-18	1.13 (17184,7213,5262,2497)
cellular process	3.68E-22	1.36E-18	1.07 (17184,11815,5262,3885)
metabolic process	2.74E-21	7.6E-18	1.12 (17184,7829,5262,2681)
macromolecule metabolic process	3.83E-18	8.5E-15	1.14 (17184,5804,5262,2025)
biological regulation	1.29E-17	2.38E-14	1.10 (17184,8262,5262,2786)

cellular metabolic process	1.75E-15	2.78E-12	1.11 (17184,7102,5262,2410)
cellular macromolecule metabolic process	2.22E-15	3.08E-12	1.14 (17184,5317,5262,1849)
macromolecule modification	1E-14	1.24E-11	1.25 (17184,1995,5262,762)
regulation of biological process	9.71E-14	1.08E-10	1.09 (17184,7815,5262,2615)
phosphorylation	1.77E-13	1.78E-10	1.44 (17184,634,5262,280)
phosphate-containing comp. metab. proc.	2.19E-13	2.03E-10	1.39 (17184,805,5262,342)
phosphorus metabolic process	2.19E-13	1.87E-10	1.39 (17184,805,5262,342)
protein phosphorylation	3.3E-13	2.62E-10	1.46 (17184,580,5262,259)
cellular protein modification process	3.43E-13	2.54E-10	1.24 (17184,1901,5262,721)
protein modification process	3.43E-13	2.38E-10	1.24 (17184,1901,5262,721)
regulation of cellular process	6.41E-12	4.19E-09	1.09 (17184,7402,5262,2470)
protein metabolic process	9.83E-12	6.07E-09	1.17 (17184,2958,5262,1061)
transmembrane receptor protein tyrosine kinase signaling pathway	9.11E-11	5.33E-08	1.44 (17184,491,5262,217)
response to stimulus	1.53E-10	8.47E-08	1.10 (17184,5621,5262,1901)
